# Supplementary material for: The interactome of intact mitochondria by cross-linking mass spectrometry provides evidence for coexisting respiratory supercomplexes
Source: Mol Cell Proteomics. 2017 Dec 8;17(2):216–32. doi: 10.1074/mcp.RA117.000470 (PMC5795388; doi:10.1074/mcp.RA117.000470)
Supplement: Supplemental Data [file supp_RA117.000470_133922_0_supp_23978_fzffwf.zip › spectra_annotation/mito_DR_spectra_annotation/499-2-6-1-16-1.pdf]

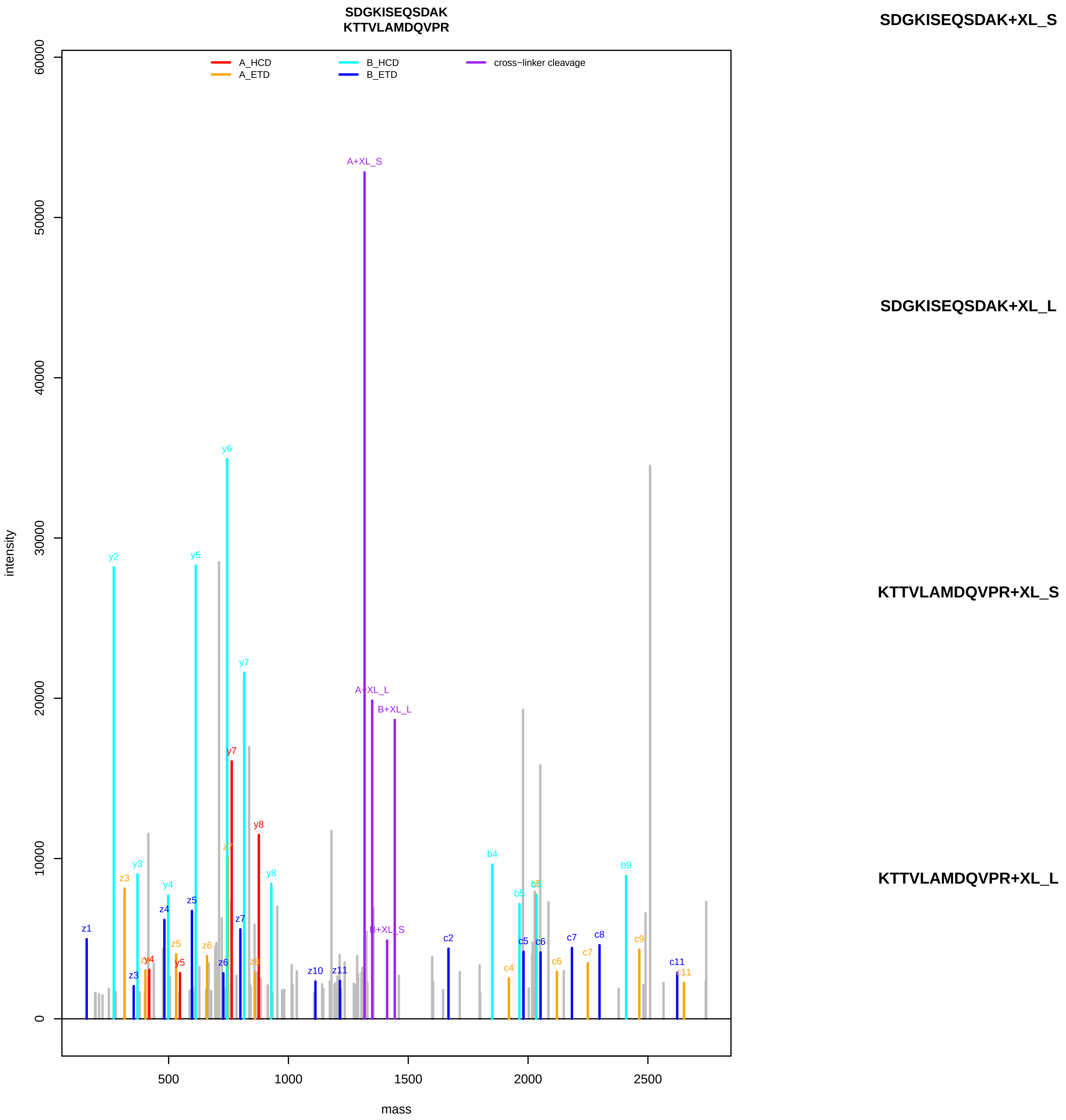
SDGKISEQSDAK
KTTVLAMDQVPR
A_HCD
A_ETD
B_HCD
B_ETD
cross−linker cleavage
intensity
mass
0
10000
20000
30000
40000
50000
60000
500
1000
1500
2000
2500
A+XL_S
A+XL_L
B+XL_L
B+XL_S
y2
y3
y4
y5
y6
y7
y8
y7
y8
y4
y5
z1
z3
z4
z5
z6
z7
z10
z11
z3
z4
z5
z6
z7
z8
b4
b5
b9
c2
c5
c6
c7
c8
c11
c4
c6
c7
c9
c11
SDGKISEQSDAK+XL_S
SDGKISEQSDAK+XL_L
KTTVLAMDQVPR+XL_S
KTTVLAMDQVPR+XL_L
